# Medication-related examination questions in clinical courses within undergraduate medical education

**DOI:** 10.1007/s00228-026-04162-z

**Published:** 2026-08-12

**Authors:** Susanna M. Wallerstedt, Lars Börjesson, Katarina Jood

**Affiliations:** 1 Department of Pharmacology, Institute of Neuroscience and Physiology, Sahlgrenska Academy, University of Gothenburg, Box 431, Gothenburg, SE-405 30 Sweden; 2 Centre for Health Technology Assessment, Sahlgrenska University Hospital, Region Västra Götaland, Gothenburg, Sweden; 3 Department of Surgery, Institute of Clinical Sciences, Sahlgrenska Academy, University of Gothenburg, Gothenburg, Sweden; 4 Department of Surgery, Sahlgrenska University Hospital, Region Västra Götaland, Gothenburg, Sweden; 5 Department of Clinical Neuroscience, Institute of Neuroscience and Physiology, Sahlgrenska Academy, University of Gothenburg, Gothenburg, Sweden; 6 Department of Neurology, Sahlgrenska University Hospital, Region Västra Götaland, Gothenburg, Sweden

**Keywords:** Examination, Medical programme, Pharmacotherapy, Prescribing, Teaching

## Abstract

**Purpose:**

Pharmacotherapy is a core competence for physicians and students’ learning is guided by examinations. This study aimed to examine medication-related examination questions across clinical courses spanning four semesters within a Swedish medical programme.

**Methods:**

In total, 375 questions used in four examinations (spring 2025; University of Gothenburg) were categorised independently, followed by consensus discussions, into mutually exclusive groups. In the first step, it was determined whether a question was medication-related; if so, the second step involved categorising its content according to the WHO 6-Step Model, which structures the physician ‘s pharmacotherapeutic workflow from defining the patient’s problem (differential diagnosis) and the therapeutic goal, to selecting and prescribing appropriate medications, counselling, and planning for monitoring and follow-up.

**Results:**

Ninety-four (25%) questions were related to medications, and an additional 44 (12%) included medications but they were not the primary focus. Half of the medication-related examination questions concerned general medication knowledge, including items where a condition was to be matched with the standard treatment (*n* = 47). The remaining questions (*n* = 47) addressed the first (*n* = 8), second (*n* = 5), third (*n* = 33), and fifth (*n* = 1) steps of the WHO 6-Step Model. No questions were related to the fourth and sixth steps.

**Conclusion:**

Pharmacotherapeutic aspects appeared as an integral component of written examinations in undergraduate clinical courses, but the questions often concerned drug selection and factual recall; other steps in the WHO Model deserve attention in other assessment procedures, including pharmacotherapy-related differential diagnosis, goal setting, technical aspects of prescribing, patient counselling, and follow-up monitoring.

## Introduction

Pharmacotherapy is a core clinical competence for physicians, encompassing professional tasks that range from identifying the patient’s problem, i.e., differential diagnosis, and defining the therapeutic goal, to selecting and prescribing appropriate medications, counselling patients, and planning for monitoring and follow-up. The structure of this workflow is reflected in the 6-Step Model issued by the World Health Organization (WHO) [1].

Representing a core competence, all medical graduates must master the fundamentals of pharmacotherapy, including the steps of the WHO 6-Step Model, which emphasises individualised pharmacotherapy rather than mere recall of medication facts and standard “first‑line” therapies that constitute general medication knowledge. Undergraduate clinical courses play a crucial role in this learning process; encountering patients during clinical placements offers valued opportunities to build on theoretical knowledge and translate theory into practice under the guidance of professionally responsible physicians [2, 3]. Furthermore, because students’ learning is guided by examinations, summative assessments are essential [4]. However, to the best of our knowledge, the pharmacotherapeutic content of examination questions in undergraduate clinical courses has not previously been explored, nor has the extent to which these questions reinforce the steps of the WHO 6-Step Model.

In this study, we examined medication-related examination questions in clinical undergraduate courses within a medical programme, with a specific focus on the extent to which they addressed the six steps of the WHO model that reflect physicians’ workflow in clinical practice.

## Methods

### Setting

In Sweden, a new six-year medical programme is currently being implemented, and the first cohort reached their eighth semester in the spring of 2025. The new programme aligns with other European curricula and grants graduates a medical license that includes prescribing rights. It replaces the previous Swedish 5.5-year programme, which required an additional 1.5-year internship before a medical license could be obtained. The programme is guided by a national framework of 23 learning outcomes for the medical degree, as specified in the Swedish Higher Education Ordinance, and implemented across the seven universities that offer medical education [5]. At the University of Gothenburg, clinical courses begin in the fifth semester, following preclinical courses in the preceding semesters.

### Design and data

In this exploratory study, we examined examination questions from the spring semester of 2025 across clinical courses in semesters five through eight of the medical programme at the University of Gothenburg. These courses covered infectious diseases, immunology, microbiology, allergology, venerology, and rheumatology (fifth semester); internal medicine, clinical pharmacology, clinical chemistry, clinical physiology, and radiology (sixth semester); surgery, orthopaedics, urology, anaesthesiology and intensive care, radiology, and oncology (seventh semester); and psychiatry, neurology, otorhinolaryngology, and ophthalmology (eighth semester). Anonymous student results from the summative assessments were extracted from the digital examination platform.

### Assessments

The assessments were performed by senior physicians specialised in clinical pharmacology (SMW), surgery (LB), or neurology (KJ), all of whom academically merited as professors and with long-standing experience as medical teachers. Two were also the director (LB) and the chair of the council (KJ) of the medical programme at the University of Gothenburg, and two had previously published pedagogic research related to the WHO 6-Step Model [3, 6].

All three assessors first independently determined whether the examination questions were medication-related. Those identified as such were further classified according to the WHO 6-Step Model: (1) Define the patient’s problem; (2) Specify the therapeutic objective; (3) Assess whether standard treatment is suitable for the specific patient (and adjust if necessary) (slightly adapted from the original phrasing: “Verify whether your P-treatment is suitable for this patient” to better align with current practice); (4) Convey adequate information to the dispenser or administrator to practically enable drug treatment (slightly rephrased from the original phrasing (“Start the treatment”) to reflect the intention of the technical aspects of prescribing, such as electronic prescriptions issued to pharmacies); (5) Give information, instructions, and warnings; and (6) Monitor (stop) the treatment. In the independent assessments, multiple steps could be selected, serving as a basis for the subsequent consensus discussions among all authors.

In the consensus discussions, discrepancies in the independent assessments were discussed in detail until consensus was reached. We first determined whether each question was medication-related. Questions were not considered medication-related if medications were not the primary focus, for example it they appeared only as distractors. In the next step, all medication-related questions were classified according to the WHO 6-Step Model using mutually exclusive categories, with each question assigned to the step most prominently reflected in its content. Medication-related questions that did not fit within the WHO model, instead targeting general medication knowledge rather than patient-specific prescribing, were categorised as “general knowledge”. Such questions typically involved the pairing of a diagnosis or clinical condition with a guideline-listed first-line medication, without requiring clinical reasoning, patient tailoring, or follow-up planning.

### Statistics

Descriptive statistical analyses were conducted using SPSS Statistics for Windows, version 27.0 (IBM Corp., Armonk, NY, USA). For exploratory purposes, we compared students’ performance on medication-related and non-medication-related examination questions by analysing the proportions of correct answers using the Mann–Whitney test. For these analyses, only single-best-answer multiple-choice questions (MCQs) were included.

## Results

Among a total of 375 examination questions, 94 (25%) were medication-related (Table 1). In an additional 44 (12%) questions, medications appeared as distractors but were not the primary focus.

**Table 1:** Medication-related examination questions, out of all examination questions (*n* = 375), in clinical courses across four semesters within undergraduate medical education

			Medication-related questions n/total (percent)
Medication-related questions	Among all questions		94/375 (25)
	By semester: specialty/specialties	5: Infectious diseases, immunology, microbiology, allergology, venerology, rheumatology	29/80 (36)
		6: Internal medicine, clinical pharmacology, clinical chemistry, clinical physiology, radiology	27/100 (27)
		7. Surgery, orthopaedics, urology, anaesthesiology and intensive care, radiology, oncology	12/75 (16)
		8. Psychiatry, neurology, otorhinolaryngology, ophthalmology	26/120 (22)
Content of medication-related questions	General knowledge^a^		47/94 (50)
	WHO step	1. Define the patient’s problem (differential diagnosis)	8/47 (17)
		2. Specify the therapeutic objective	5/47 (11)
		3. Standard treatment suitable? Adjust!^b^	33/47 (70)
		4. Convey information to administering/dispensing professionals^c^	0^d^
		5. Give information, instructions, and warning	1/47 (2)
		6. Monitor (stop) the treatment	0^d^

WHO = World Health Organization

^a^Including general questions about medications as well as rote learning questions where a condition was to be combined with a standard treatment, i.e., questions not requiring clinical reasoning, patient-tailoring, or follow-up planning

^b^Slightly rephrased from the original wordings (“Verify whether your P-treatment is suitable for this patient”) to better align with current practice

^c^Slightly rephrased from the original wording (“Start the treatment”) to reflect the intention of the technical aspects of prescribing, such as electronic prescriptions issued to pharmacies

^d^No examination questions concerned this WHO step

Half of the medication-related examination questions concerned general knowledge about medications (*n* = 47; 13% of all questions). The remaining medication-related questions primarily addressed the third step of the WHO 6-step model (*n* = 33; 9% of all questions). No questions addressed steps 4 or 6 in the WHO model.

In total, 325 (87%) examination questions were MCQs. These were answered by 469 students across four semesters of clinical courses. Exploratory analyses revealed no statistically significant differences in performance between medication-related questions and non-medication-related questions (Table 2).

**Table 2:** Students’ results on medication-related MCQs and other MCQs, in examinations of clinical courses across four semesters within a medical programme

		Number of students	Number of MCQs/total	Percentage correct answers, median (IQR)		*P*-value
				Medication-related *(number of MCQs)*	Non-medication-related *(number of MCQs)*	
All		469	325/375^a^	82 (64‒94) (*78*)	85 (66‒94) (*247*)	0.36
By semester: specialty/specialties	5: Infectious diseases, immunology, microbiology, allergology, venerology, rheumatology	143	30/80^a^	70 (63‒87) (*13*)	84 (71‒94) (*17*)	0.18
	6: Internal medicine, clinical pharmacology, clinical chemistry, clinical physiology, radiology	133	100/100	72 (65‒93) (*27*)	82 (67‒92) (*73*)	0.40
	7. Surgery, orthopaedics, urology, anaesthesiology and intensive care, radiology, oncology	100	75/75	81 (64‒98) (*12*)	76 (47‒94) (*63*)	0.40
	8. Psychiatry, neurology, otorhinolaryngology, ophthalmology	93	120/120	88 (58‒96) (*26*)	88 (71‒96) (*94*)	0.80

IQR = interquartile range, MCQ = multiple-choice question

^a^50 questions were not MCQs (45 modified essay questions and 5 short answer questions)

## Discussion

This study shows that approximately one in four examination questions in clinical undergraduate courses within a medical programme addresses pharmacotherapy. Half of the pharmacotherapy-related questions concern general medication knowledge that is not explicitly linked to physicians’ professional duties in treating individual patients. The remaining half engage some, but not all, steps of the WHO 6-Step Model, with substantial emphasis on the third step: determining an appropriate treatment for a specific patient.

Our results illustrate that pharmacotherapy is an integral component of examinations in undergraduate clinical courses. Notably, 13% of all examination questions, i.e., approximately one in eight questions, directly addressed the physician’s workflow in treating patients with medications, requiring at least some degree of clinical reasoning, individualisation, or follow-up planning. For comparison, we also categorised examination questions from the preclinical pharmacology course in the fourth semester: all were medication-related and classified as general knowledge (data not shown). Examinations in undergraduate clinical courses therefore represent, at least to some extent, a progression from the preclinical setting. One may speculate that these findings indicate a potential for programmatic assessment, i.e., a model of longitudinal competency assessments throughout the curriculum, designed not only to inform critical decisions but also to support learning and development [7]. Indeed, this approach has been described as appealing but organisationally demanding within pharmacotherapy-related education across the undergraduate curriculum [8].

The findings of this study suggest that written examinations often prioritise drug selection and factual recall, the foundation for therapy scripts in therapeutic reasoning [9], over other essential steps of the pharmacotherapeutic process, such as goal setting, safe prescription writing, patient counselling, and follow-up monitoring. Although not evaluated in the present study, it could be argued that other summative approaches, such as objective structured clinical examinations (OSCEs) and evaluations of entrusted professional activities [10] during workplace‑based assessments [8] may address these steps. Indeed, summative assessments in pharmacotherapy may be particularly important, as they have been reported to yield higher scores than purely formative assessments [11]. Nevertheless, the limited coverage of some critical pharmacotherapeutic steps within written examination questions, representing essential competencies for rational and person-centred prescribing, underscores the need to actively incorporate these components into other educational assessments. To ensure balanced coverage of the WHO steps, test blueprinting, defined as a structured mapping of assessment items to learning objectives and content areas [12], could be instrumental.

It may not be surprising that Step 4, which includes prescribing correctly within electronic systems, was not represented in the examination questions. Although virtually all prescribing in Sweden is performed electronically, multiple electronic health record (EHR) systems are in use, each with its own interface and functionalities. These systems also integrate clinical decision support in different ways and to varying extents, such as alerts for drug-drug interactions, dosing guidance for patients with impaired renal function or paediatric patients, and warnings about potentially inappropriate medications (PIMs) in older people. However, such alerts pose a well-recognised risk of alert fatigue; only a minority of drug interaction alerts warrant clinical action [13], and similar findings have been reported for PIMs [14]. Therefore, the fourth step of the WHO model is likely best taught and assessed through workplace-based training. Indeed, training in distinguishing essential from non-essential information is crucial, as it helps prepare medical students to determine what is relevant for each individual patient. In this context, it is also worth noting that the design of EHR systems has been linked to both usability and medication safety [15].

Interestingly, fewer than one in ten medication-related examination questions, and about one in fifty questions overall, addressed the first step of the WHO 6-Step Model, which involves defining the patient’s problem. Because adequate differential diagnosis, including consideration of whether adverse drug reactions may contribute to the patient’s condition, is a prerequisite for all subsequent steps, including therapeutic reasoning [9], our findings suggest that this first step warrants greater emphasis in examination questions within undergraduate clinical courses.

While promoting adherence in the fifth step explicitly relates to establishing a good physician–patient relationship, other aspects of this step involve general medication knowledge, such as the maximum allowed dose, how the drug should be taken, when it should not be taken, and which adverse effects may occur. To minimise the risk that these latter elements are overlooked in theoretical examinations, it could be argued that the fifth step could also be reflected in examination questions in preclinical courses. Indeed, although three large European randomised controlled trials [16–18] have not demonstrated patient or healthcare benefits when pharmaceutical-level knowledge is provided by other professionals during parts of the physician prescribing process, explicitly requiring such general knowledge from graduating physicians may still be considered reasonable.

The results of our exploratory analysis of student performance are encouraging, as they suggest that medication-related questions may be prioritised for learning by medical students to a similar extent as other questions in undergraduate clinical courses. However, it should be noted that the MCQ format may have limitations in capturing students’ competence [19], and that effective assessments of pharmacotherapeutic competence therefore remain critical. Indeed, although estimates of medication-related harm vary greatly depending on perspective [20] and often do not take the benefit–risk balance of pharmacotherapy into account [21], medication-related hospitalisations and deaths that are preventable still occur [22, 23].

Strengths of this study include the use of a multi-course sample across several clinical specialities and the explicit mapping to the established, practice‑oriented WHO 6-Step Model. Additionally, the categorisation procedure, consisting of independent classifications followed by consensus discussions among experienced medical teachers in the specialties of clinical pharmacology, surgery, and neurology, can be considered a further strength. Limitations include the single-university setting, which restricts generalisability; curricular differences across institutions may result in different distributions. Nevertheless, examining medication-related questions in clinical undergraduate courses in relation to physicians’ workflow may inspire similar evaluations in other settings. A further limitation to generalisability is that only examination questions up to the eighth semester were included, as the first student cohort in the new six-year medical programme reached this stage in spring 2025. Consequently, questions from paediatrics and gynaecology, scheduled for the ninth semester, were not analysed. The remaining semesters (ten to twelve) primarily consist of a master’s thesis, elective courses, and clinical training within internal medicine, surgery, geriatrics, and family medicine. Another limitation is that only questions from the overall concluding semester examinations were included, whereas the auto‑graded digital medication quizzes implemented on the learning platform in the eighth semester, used for both practice and summative assessment and based on the WHO six‑step model [3], were not considered. Furthermore, analysing data at the semester level, as the examinations are structured in practice, does not allow conclusions regarding specialty-specific differences; larger datasets would be required for such analyses. Finally, step assignment was based on identifying a single dominant step; because some questions arguably involved several steps, this approach may have resulted in undercounting certain steps. However, we consider the absence of questions in WHO steps 4 and 6 to reflect a true gap.

In conclusion, pharmacotherapeutic aspects appear to be an integral component of written examinations in undergraduate clinical courses. Our findings, including that most medication-related examination questions focus either on general knowledge not specific to the professional prescribing role or on selecting an appropriate treatment for a specific patient, suggest that teachers and examiners need to explicitly integrate key prescribing responsibilities into other assessment formats. This is necessary to ensure that graduating medical students develop competence across the full continuum of physicians’ pharmacotherapeutic practice in clinical care, including pharmacotherapy-related differential diagnosis, goal setting, technical aspects of prescribing, patient counselling, and follow-up monitoring. Incorporating test blueprinting may help ensure balanced coverage of the WHO steps in examination questions.

## Data Availability

The datasets generated and analysed during the current study are available upon reasonable request to the corresponding author.
